# Homologous Recombination Deficiency Assays in Epithelial Ovarian Cancer: Current Status and Future Direction

**DOI:** 10.3389/fonc.2021.675972

**Published:** 2021-10-14

**Authors:** Ying-Cheng Chiang, Po-Han Lin, Wen-Fang Cheng

**Affiliations:** ^1^ Department of Obstetrics and Gynecology, College of Medicine, National Taiwan University, Taipei, Taiwan; ^2^ Department of Medical Genetics, National Taiwan University Hospital, Taipei, Taiwan; ^3^ Graduate Institute of Medical Genomics and Proteomics, College of Medicine, National Taiwan University, Taipei, Taiwan; ^4^ Graduate Institute of Clinical Medicine, College of Medicine, National Taiwan University, Taipei, Taiwan; ^5^ Graduate Institute of Oncology, College of Medicine, National Taiwan University, Taipei, Taiwan

**Keywords:** homologous recombination deficiency, epithelial ovarian cancer, PARP inhibitor, genomic scar, RAD51 foci formation, mutational signatures

## Abstract

Epithelial ovarian cancer (EOC) patients are generally diagnosed at an advanced stage, usually relapse after initial treatments, which include debulking surgery and adjuvant platinum-based chemotherapy, and eventually have poor 5-year survival of less than 50%. In recent years, promising survival benefits from maintenance therapy with poly(ADP-ribose) polymerase (PARP) inhibitor (PARPi) has changed the management of EOC in newly diagnosed and recurrent disease. Identification of *BRCA* mutations and/or homologous recombination deficiency (HRD) is critical for selecting patients for PARPi treatment. However, the currently available HRD assays are not perfect predictors of the clinical response to PARPis in EOC patients. In this review, we introduce the concept of synthetic lethality, the rationale of using PARPi when HRD is present in tumor cells, the clinical trials of PARPi incorporating the HRD assays for EOC, the current HRD assays, and other HRD assays in development.

## Introduction

**Figure 1:**
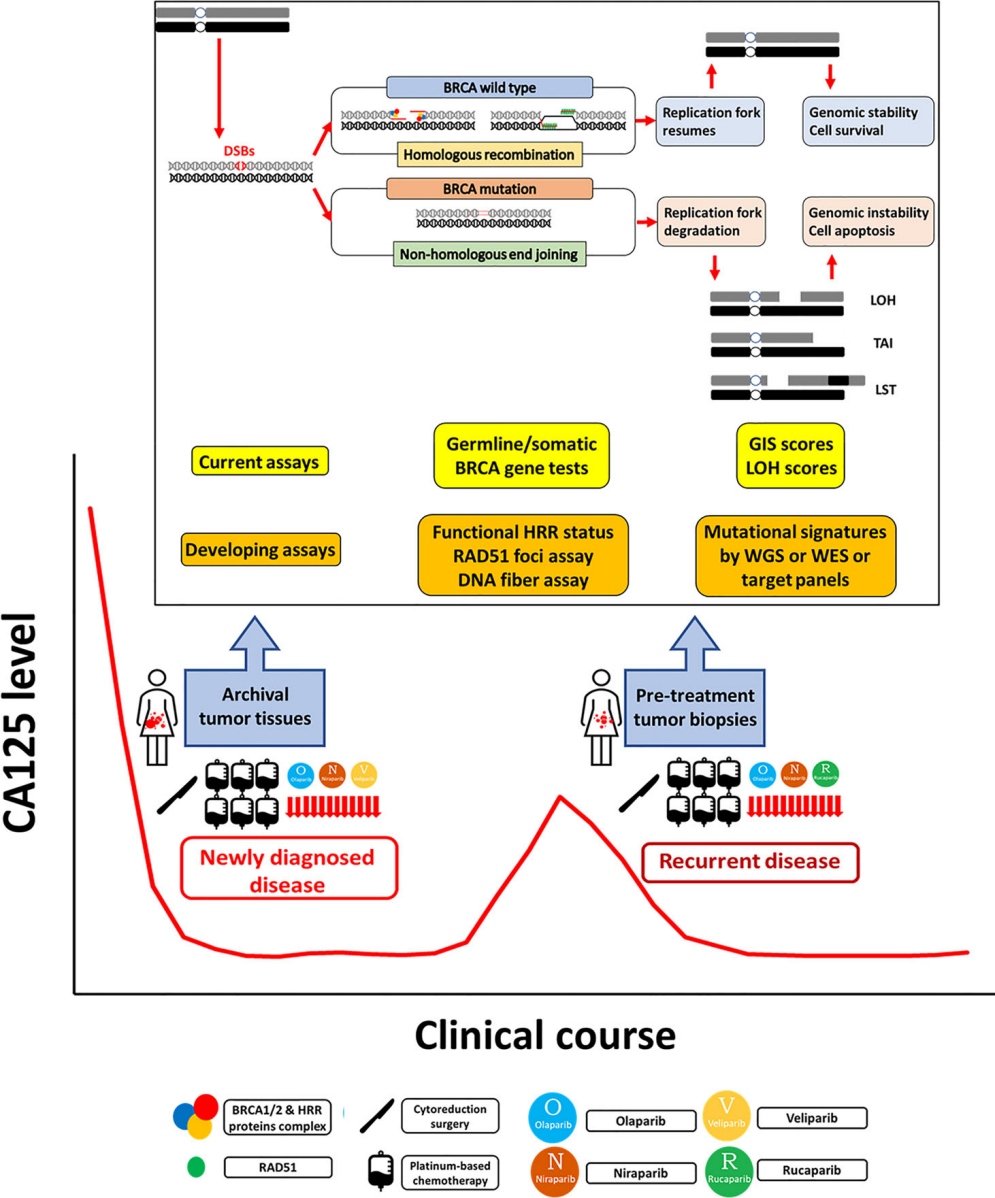
Homologous recombination deficiency assays in epithelial ovarian cancer. Homologous recombination repair (HRR) is an important pathway for repairing DNA double-strand breaks (DSBs). In HRR, DSBs are detected and bound by BRCA1/2 and other HRR protein complexes to localize RAD51 to the exposed single-strand DNA, which then invades the DNA sequence on a homologous sister chromatid to synthesize the new DNA strand. Homologous recombination deficiency (HRD) occurs when HRR is impaired, especially by BRCA mutation. DSBs are repaired by other error-prone repair pathways, including nonhomologous end joining (NHEJ), which may cause point mutations, small insertions or deletions, and even large-scale chromosomal rearrangements in the repaired DNA strands. In the S phase of the cell cycle, the replication forks stop when encountering DNA damage to allow DNA repair before replication continues. After the damage is repaired, the stalled fork resumes replication. If the damage cannot be repaired, the stalled replication fork undergoes fork collapse, comprising fork degradation to cell apoptosis. In general, HRD assays have three main categories: germline or somatic mutations of genes in the HRR pathway, genomic scars or mutational signatures representing the patterns of genomic instability, and checking the functional status of HRR. Germline and somatic BRCA tests should be performed in all patients with newly diagnosed epithelial ovarian cancer (EOC). The current HRD assays based on SNP-based microarray technologies measure loss of heterozygosity (LOH), telomeric allelic imbalance (TAI), and large-scale state transitions (LSTs). The genomic instability score (GIS) combines the information derived from the LOH, TAI, and LST to represent the degree of genomic instability. Functional assays of HRR status include RAD51 foci assays and DNA fiber assays. The RAD51 subnuclear foci is generally present after DNA damage in normal cells, but HRD cells cannot form RAD51 foci. The DNA fiber assay was developed to evaluate the dynamics of the replication fork. Mutational signatures describe distinct patterns of nucleotide transitions with the surrounding nucleotide context from next-generation sequencing (NGS) data for human tumors. The developing comprehensive genomic scar assays include whole genome sequencing (WGS), whole exome sequencing (WES), and target gene panels consisting of variable sizes of selected cancer-susceptible genes. PARPi maintenance therapy benefits newly diagnosed advanced stage and platinum-sensitive recurrent EOC patients. The current genomic scar-based HRD assays can identify additional wild-type BRCA patients who may benefit from PARPi therapy. However, some issues need to be resolved. For tissue retrieval, multistep sampling to obtain archival and pretreatment tumor tissues over the clinical course has the potential to guide the therapy. For precision medicine, it is ideal to develop a comprehensive model to integrate clinical platinum sensitivity, genomic scar/mutational signatures, and functional tests to provide both past evidence of HRD and the current functional ability of HRR.

Epithelial ovarian cancer (EOC) is a major cause of death in women worldwide (1–5). Due to a lack of specific symptoms and biological markers for early diagnosis, most ovarian cancer patients are diagnosed at an advanced stage in which the disease has spread beyond the pelvis, with an associated 5-year survival of less than 50% (6). The primary standard treatment, debulking surgery, and adjuvant chemotherapy with a platinum and paclitaxel regimen, can achieve good initial response rates, but the majority of ovarian cancer patients eventually relapse (7). Based on the evidence to date, antiangiogenic agents and poly-adenosine diphosphate ribose polymerase (PARP) inhibitors (PARPis) are the most promising targeted therapies for EOC in the past decade (8, 9). Maintenance therapy with PARPis has rewritten the management of EOC in newly diagnosed and recurrent disease (10–15). In the era of precision medicine, it is important to select the appropriate patients to benefit from the targeted therapy. Evaluating homologous recombination deficiency (HRD) in tumor cells as a potential predictor of the response to a PARPi is an important clinical issue. Identification of *BRCA* mutations and/or HRD status in clinical specimens is critical to selecting EOC patients for PARPi treatment, which has been evaluated in several clinical trials (10–15). The US Food and Drug Administration (FDA) has also approved companion diagnostic tests for PARPi use based on these trials. Cost-effective analysis has shown that PARPi therapy should preferably be reserved for HRD-positive EOC patients until the cost is significantly reduced (16, 17). The measurement of HRD is important for the appropriate use of PARPis in EOC patients, and understanding the various HRD assays will aid clinical practice (18). The HRD and HRD assay terminology should be intrepreted with caution as it might appear to be somewhat confusing (**Table 1**). In general, HRD is different from HRD test positive. HRD test positive means that the tumors or patients have deficiency of homologous recombination repair pathway, including those with germline and somatic *BRCA* mutations. HRD test negative actually means that the tumors or patients have intact homologous recombination repair pathway, indicating homologous recombination repair (HRR) proficient. However, the currently available HRD assays are not perfect predictors of the PARPi response because, in previous trials, HRD-positive and HRD-negative patients defined by the current assays both benefited from PARPi. In this review, we introduce the concept of synthetic lethality, the rationale of using PARPis when HRD is present in tumor cells, the clinical trials of PARPis incorporating the HRD assays for EOC, the current HRD assays, and other HRD assays in development (**Figure 1**).

**Table 1:** Terminology of homologous recombination.

HRR	Homologous recombination repair	An important error-proof DNA damage repair pathway to restore the original sequence at DNA double-strand break site generally in S and G2 phases of cell cycle by homologous recombination. In the process, DNA double-strand breaks are detected by MRE11-RAD50-NSB1 complex, which in turn recruit ATM and BRCA1. Then, a small part of the DNA sequence at the DNA double-strand break site is removed to expose the single-strand DNA. Through BRCA1, BRCA2, and PALB2 localizing to the exposed single-strand DNA, RAD51 binds to the single-strand DNA and invades the DNA sequence on a homologous sister chromatid, which is used as a template for synthesizing the new DNA stand.
HRP	Homologous recombination proficient	The cells are able to repair DNA damage, especially DNA double-strand breaks, by homologous recombination repair pathway to preserve the original genetic information.
HRD	Homologous recombination deficiency	The condition that homologous recombination repair pathway is impaired in the cells and the DNA double-strand breaks are repaired by another error-prone repair pathways, such as nonhomologous end joining (NHEJ), microhomology-mediated end joining or single-strand annealing, which may cause point mutations, small insertions or deletions, and even large-scale chromosomal rearrangements in the repaired DNA strands. The HRD can be defined by various HRD assays in clinical trials, and the consensus of the cutoff value of the various assays to define HRD is needed to be determined.
HRD positive	Homologous recombination deficiency positive	HRD positive by a HRD assay indicates that the tumors are deficient in the error-proof homologous recombination repair pathway, and the DNA double-strand breaks are predominantly repaired by other error-prone repair pathways.
HRD negative	Homologous recombination deficiency negative	HRD negative by a HRD assay indicates that the tumors are proficient in the error-proof homologous recombination repair pathway, and the DNA double-strand breaks are predominately repaired by homologous recombination repair pathway. The “HRD negative” is synonymous with HR proficient or HR competent.

## Synthetic Lethality

DNA damage in cells may result from exogenous or endogenous sources, such as oxidative damage, radiation, ultraviolet light, cytotoxic materials, and replication errors, among others (19). Accumulation of unrepaired DNA damage is harmful to cells, leading to genomic instability and, eventually, apoptosis. Several DNA damage response (DDR) pathways are present in cells to fix single-strand breaks (SSBs) or double-strand breaks (DSBs) in the damaged DNA. Dysregulation of the DDR during the cell cycle is associated with carcinogenesis (20). HRR is an important DDR pathway for the repair of DNA DSBs that generally acts in the S and G2 phase of the cell cycle. HRR is an error-proof repair mechanism in which the original sequence at the DSB site is restored by homologous recombination. In HRR, DSBs are detected and bound by the MRE11-RAD50-NSB1 (MRN) complex, which in turn recruits ATM and BRCA1. A small part of the DNA sequence at the DSB site is removed to expose the single-stranded DNA. With localization of BRCA1, BRCA2, and PALB2 at the exposed DNA, the DNA recombinase RAD51 binds to the single-stranded DNA and invades the DNA sequence on a homologous sister chromatid, which is used as a template for synthesizing the new DNA strand, effectively preserving the original genetic information (21, 22). Homologous recombination deficiency (HRD) occurs when HRR is impaired and DSBs are repaired by another, error-prone repair pathway, such as nonhomologous end joining (NHEJ), microhomology-mediated end joining, or single-strand annealing, which may cause point mutations, small insertions or deletions, and even large-scale chromosomal rearrangements in the repaired DNA strands (23–25).

PARP plays multiple roles in repairing both SSBs and DSBs, such as binding to DNA breaks, recruiting repair proteins, and resolving collapsed replication forks (26–30). The small molecule inhibitors of PARP (i.e., PARPis) trap PARP at the sites of SSBs, stalling the replication fork. When the stalling replication fork is encountered by the DNA replication machinery, it creates a DSB. The PARPi-induced DSB undergoes HRR in normal cells. However, in cells with HRD, PARPi-induced DSBs are repaired by the error-prone pathways, leading to genomic instability, apoptosis, and cell death, the so-called synthetic lethality (26–31). The development of PARPis for clinical management is based on the increasing sensitivity of BRCA-mutated cancer cells to PARPis (32, 33). Cancers with HRD are also more sensitive to platinum drugs and topoisomerase inhibitors (26, 34). Although platinum sensitivity has been considered a marker of PARPi sensitivity, the correlation is unsatisfactory (35). In contrast, homologous recombination-proficient (HRP) cells are often resistant to PARPis or platinum drugs.

## Clinical Trials of PARP Inhibitors in Relapsed or Newly Diagnosed Ovarian Cancer

DDR pathways are a potential target for cancer therapy based on the concept of synthetic lethality, with the aim of specifically killing cancer cells dependent on a compensatory DNA repair pathway for survival (20). In recent decades, several phases II and III clinical trials have demonstrated the survival benefits of PARPi use in the treatment of EOC, especially high-grade serous type, in recurrent and newly diagnosed disease. In these trials, *BRCA* gene mutation tests and/or HRD assays were investigated using a primary study design or retrospective exploratory analysis to determine the predictive value of these assays in stratifying EOC patients that benefit from PARPi therapy. **Table 2** summarizes several important trials of PARPis in the setting of recurrent and newly diagnosed disease.

**Table 2:** Clinical trials of PARPi in recurrent or newly diagnosed epithelial ovarian cancer (EOC).

Recurrent EOC, multiple prior lines of therapy	PARPi	Assays (genetic biomarkers)	Analysis subgroups	PARPi		
				*n*	ORR	DoR or PFS (months)
Study 42 (EOC *n* = 193) (NCT01078662)	Olaparib	Local BRCA gene test (gBRCA mutation)	ITT (gBRCAmut)	193	31.1%	7
			gBRCAmut, ≥3 prior lines of chemotherapy and measurable disease	137	34%	7.9
ARIEL2 (part 1) (*n* = 204) (NCT01891344)	Rucaparib	Foundation Focus CDx BRCA LOH assay	HRD-p (g/sBRCAmut)	40	85%	12.8
			HRD-p (BRCAwt and LOH-high)	82	44%	5.7
			HRD-n (BRCAwt and LOH-low)	70	20%	5.2
			BRCAwt and LOH unclassified	12	58%	N.A.
QUADRA (*n* = 463) (NCT02354586)	Niraparib	Germline BRCA testing Myriad myChoice HRD	All	463	28%	5.5
			BRCA mutated	63	29%	9.2
			HRD positive	189	15%	9.2
			HRD negative or unknown	230	3%	10.1
SOLO3 (*n* = 266) (NCT00628251)	Olaparib *vs*. nonplatinum chemotherapy	Germline BRCA mutation by Myriad testing	Olaparib in patients with measurable disease	151	72.2%	N.A.
			Chemotherapy in patients with measurable disease	72	51.4%	N.A.
			Olaparib in patients with ≥2 prior lines of treatment	121	84.6%	13.4
			Chemotherapy in patients with ≥2 prior lines of treatment	59	61.5%	9.2
Recurrent EOC, platinum-sensitive	PARPi	Assays (genetic biomarkers)	Analysis subgroups	PFS (months)		
				PARPi	Placebo	HR (95% CI)
Study19 (*n* = 265) (NCT00753545)	Olaparib *vs*. placebo	Foundation Medicine (tBRCA mutation)	ITT (all patients)	10.8 (*n* = 136)	5.4 (*n* = 129)	0.35 (0.25–0.49)
			HRD-p (BRCAmut)	11.2 (*n* = 74)	4.3 (*n* = 62)	0.18 (0.1–0.31)
			HRD-n (BRCAwt)	7.4 (*n* = 57)	5.5 (*n* = 61)	0.54 (0.34–0.85)
NOVA (*n* = 553) (NCT01847274)	Niraparib *vs*. placebo	Myriad BRACAnalysis test (gBRCA mutation), myChoice^®^ HRD (GIS)	HRD-p (gBRCAmut)	21 (*n* = 138)	5.5 (*n* = 65)	0.27 (0.17–0.41)
			HRD-p (gBRCAwt and GIS ≥42)	12.9 (*n* = 106)	3.8 (*n* = 56)	0.38 (0.24–0.59)
			HRD-n (gBRCAwt)	9.3 (*n* = 234)	3.9 (*n* = 116)	0.45 (0.34–0.61)
			HRD-n (gBRCAwt and GIS <42)	6.9 (*n* = 92)	3.8 (*n* = 42)	0.58 (0.36–0.92)
SOLO2 (*n* = 295) (NCT01874353)	Olaparib *vs*. placebo	Myriad BRACAnalysis test (gBRCA mutation)	ITT (gBRCAmut)	19.1 (*n* = 196)	5.5 (*n* = 99)	0.30 (0.22–0.41)
ARIEL3 (*n* = 564) (NCT01968213)	Rucaparib *vs*. placebo	Foundation Medicine T5 NGS assay (g/s BRCA mutation, 28 HRR gene mutation, LOH)	ITT (all patients)	10.8 (*n* = 375)	5.4 (*n* = 189)	0.36 (0.3–0.45)
			HRD-p (g/sBRCAmut)	16.6 (*n* = 130)	5.4 (*n* = 66)	0.23 (0.16–0.34)
			HRD-p (g/sBRCAmut or LOH-high)	13.6 (*n* = 236)	5.4 (*n* = 118)	0.32 (0.24–0.42)
			HRD-n (BRCAwt)	8.2 (*n* = 245)	5.4 (*n* = 123)	0.44 (0.34–0.56)
			HRD-p (BRCAwt and LOH-high)	9.7 (*n* = 106)	5.4 (*n* = 52)	0.44 (0.29–0.66)
			HRD-n (BRCAwt and LOH-low)	6.7 (*n* = 107)	5.4 (*n* = 54)	0.58 (0.4–0.8)
Newly diagnosed EOC	PARPi	Assays (genetic biomarkers)	Analysis subgroups	PFS (months)		
				PARPi	Placebo	HR (95% CI)
SOLO1 (*n* = 391) (NCT01844986)	Olaparib *vs*. placebo	Myriad (gBRCA mutation)	ITT (gBRCAmut)	NR (*n* = 260)	13.8 (*n* = 131)	0.3 (0.23–0.41)
PRIMA (*n* = 733) (NCT02655016)	Niraparib *vs*. placebo	Myriad myChoice^®^ HRD Plus assay (tBRCA mutation and GIS)	ITT (all patients)	13.8 (*n* = 487)	8.2 (*n* = 246)	0.62 (0.5–0.76)
			HRD-p (tBRCAmut)	22.1 (*n* = 152)	10.9 (*n* = 71)	0.4 (0.27–0.62)
			HRD-p (tBRCAmut or GIS ≥42)	21.9 (*n* = 247)	10.4 (*n* = 126)	0.43 (0.31–0.59)
			HRD-p (tBRCAwt and GIS ≥42)	19.6 (*n* = 95)	8.2 (*n* = 55)	0.5 (0.31–0.83)
			HRD-n (tBRCAwt and GIS <42)	8.1 (*n* = 169)	5.4 (*n* = 80)	0.68 (0.49–0.94)
VELIA (*n* = 1140) (NCT0247058)	PTV + veliparib *vs*. PT + veliparib *vs*. PT + placebo	Myriad myChoice^®^ HRD Plus assay (tBRCA mutation and GIS)	ITT (all patients)	23.5 (*n* = 382)	17.3 (*n* = 375)	0.68 (0.56–0.83)
			HRD-p (tBRCAmut)	34.7 (*n* = 108)	22 (*n* = 92)	0.44 (0.28–0.68)
			HRD-p (tBRCAmut or GIS ≥33)	31.9 (*n* = 214)	20.5 (*n* = 207)	0.57 (0.43–0.76)
			HRD-p (tBRCAwt and GIS ≥33)	15.0 (*n* = 120)	11.5 (*n* = 130)	0.74 (0.52–1.06)
			HRD-n (tBRCAwt)	18.2 (*n* = 245)	15.1 (*n* = 254)	0.8 (0.64–1.00)
			HRD-n (tBRCAwt and GIS <33)	15.0 (*n* = 125)	11.5 (*n* = 124)	0.81 (0.6–1.09)
PAOLA-1 (*n* = 806) (NCT02477644)	Olaparib + bevacizumab *vs*. placebo + bevacizumab	Myriad myChoice^®^ HRD Plus assay (tBRCA mutation and GIS)	ITT (all patients)	22.1 (*n* = 537)	16.6 (*n* = 269)	0.59 (0.49–0.72)
			HRD-p (tBRCAmut)	37.2 (*n* = 157)	21.7 (*n* = 80)	0.31 (0.2–0.47)
			HRD-p (tBRCAmut or GIS ≥42)	37.2 (*n* = 255)	17.7 (*n* = 132)	0.33 (0.25–0.45)
			HRD-p (tBRCAwt and GIS ≥42)	28.1 (*n* = 97)	16.6 (*n* = 55)	0.43 (0.28–0.66)
			HRD-n (tBRCAwt)	18.9 (*n* = 380)	16 (*n* = 189)	0.71 (0.58–0.88)
			HRD-n (GIS <42/unknown)	16.9 (*n* = 283)	16 (*n* = 134)	0.92 (0.72–1.17)

BRCAmut, BRCA mutation; BRCAwt, BRCA wild-type; gBRCAmut, germline BRCA mutation; gBRCAwt, germline BRCA wild type; g/sBRCAmut, germline/somatic BRCA mutation; tBRCA mutation, tumor BRCA mutation; CI, confidence interval; GIS, genomic instability score; HR, hazard ratio; HRD-p, homologous recombination deficiency-positive; HRD-n, homologous recombination deficiency-negative; HRR, homologous recombination repair; ITT, intention to treat; LOH, loss of heterogeneity; ORR, objective response rate; DoR, duration of response; PARPi, PARP inhibitor; PFS, progression-free survival.

### Salvage Monotherapy With PARP Inhibitor in Recurrent EOC Patients After Multiple Prior Lines of Therapy

In the phase II study 42 trial (36, 37), the objective response rate (ORR) to olaparib in patients with germline *BRCA1/2* (g*BRCA1/2*)-mutated advanced ovarian cancer who had received ≥3 prior lines of chemotherapy was 34% (95% CI, 26–42) and median duration of the response (DoR) was 7.9 months (95% CI, 5.6–9.6). The median DoR for platinum-sensitive and platinum-resistant disease was 8.2 months (95% CI, 5.6–13.5) and 8.0 months (95% CI, 4.8–14.8), respectively. These findings suggest that olaparib monotherapy has antitumor activity in patients with g*BRCA1/2*-mutated advanced ovarian cancer following more than three prior lines of chemotherapy.

In the phase II ARIEL2 (Part 1) trial (38), the status of homologous recombination was determined by the Foundation Focus CDx BRCA loss of heterozygosity (LOH) assay. HRD was defined as more than 14% genomic LOH based on the TCGA dataset. The median progression-free survival (PFS) after rucaparib treatment was 12.8 months in the *BRCA*-mutated subgroup, 5.7 months in the LOH-high subgroup, and 5.2 months in the LOH-low subgroup. PFS was significantly longer in the *BRCA*-mutated (HR = 0.27; 95% CI, 0.16–0.44; *p* < 0.0001) and LOH-high (HR = 0.62; 95% CI, 0.42–0.90; *p* = 0.011) subgroups compared with the LOH-low subgroup. Thus, ARIEL2 (Part 1) concluded that rucaparib provides significant PFS benefits in platinum-sensitive relapsed EOC patients with *BRCA*-mutated or *BRCA* wild-type LOH-high. The findings suggest that tumor LOH can be used to identify platinum-sensitive EOC patients with *BRCA* wild-type who might benefit from rucaparib. The LOH threshold was adjusted to ≥16% in a *post-hoc* analysis to improve the selection of patients who will benefit from rucaparib (39).

In the phase II QUADRA trial (40), a clinical benefit of niraparib was observed in heavily pretreated EOC patients (median of four previous lines of therapy), including platinum-sensitive, platinum-resistant, and platinum-refractory patients. In *BRCA*-mutated patients, niraparib was more active in patients who were platinum sensitive to last line platinum-based chemotherapy (39%) compared with platinum-resistant (33%) and platinum-refractory (19%) patients. In the *BRCA* wild-type HRD-positive cohort, 20% of platinum-sensitive patients achieved a response, but only 2.4% of platinum-resistant and refractory patients had a response similar to the HRD-negative or unknown cohort (3%) (40–42).

In the phase III SOLO3 trial (43), monotherapy olaparib resulted in significant and clinically relevant improvements in ORR (72.2% *vs*. 51.4%) and PFS (13.4 *vs*. 9.2 months) compared with nonplatinum chemotherapy in patients with g*BRCA*-mutated platinum-sensitive recurrent EOC patients with at least two prior lines of platinum-based chemotherapy.

### Second-Line Maintenance Monotherapy With PARP Inhibitor in Platinum-Sensitive Recurrent EOC Patients

In the phase II study 19 trial (44), the median PFS was significantly longer in the olaparib group than in the placebo group among the *BRCA*-mutated cohort (11.2 *vs*. 4.3 months; HR = 0.18; 95% CI, 0.10–0.31; *p* < 0.0001) and *BRCA* wild-type cohort (7.4 *vs*. 5.5 months; HR = 0.54; 95% CI, 0.34–0.85; *p* = 0.0075). Study 19 concluded that platinum-sensitive recurrent serous ovarian cancer patients with a *BRCA* mutation receive the greatest benefits from olaparib maintenance therapy.

In the phase III NOVA trial (13), the status of homologous recombination was determined by the *BRCA* mutation or myChoice assay with a genomic instability score (GIS) ≥42. The median PFS was significantly longer in the niraparib group than in the placebo group among the g*BRCA*-mutated cohort (21.0 *vs*. 5.5 months; HR = 0.27; 95% CI, 0.17–0.41; *p* < 0.001), the non-g*BRCA*-mutated HRD-positive cohort (12.9 *vs*. 3.8 months; HR = 0.38; 95% CI, 0.24–0.59; *p* < 0.001), and the non-g*BRCA*-mutated cohort (9.3 *vs*. 3.9 months; HR = 0.45; 95% CI, 0.34–0.61; *p* < 0.001). NOVA concluded that the median PFS was significantly longer among the platinum-sensitive recurrent EOC patients receiving niraparib maintenance therapy regardless of the g*BRCA* mutation or HRD status.

In the phase III SOLO2 trial, the median PFS was significantly longer in the olaparib group than in the placebo group among the *BRCA*-mutated cohort (19.1 *vs*. 5.5 months; HR = 0.30; 95% CI, 0.22–0.41; *p* < 0.0001) (15). The median overall survival was significantly longer in the olaparib group than in the placebo group among the *BRCA*-mutated cohort (51.7 *vs*. 38.8 months; HR = 0.74; 95% CI, 0.54–1.00; *p* = 0.054) (45). SOLO2 concluded that olaparib maintenance therapy provides significant PFS benefits and a median overall survival benefit of 12.9 months compared with placebo in platinum-sensitive recurrent EOC patients with *BRCA* mutation.

In the phase III ARIEL 3 trial (14), the status of homologous recombination was determined by the Foundation Focus CDx BRCA LOH assay with the threshold ≥16% genomic LOH. The median PFS was significantly longer in the rucaparib group than in the placebo group among the *BRCA*-mutated cohort (16.6 *vs*. 5.4 months; HR = 0.23; 95% CI, 0.16–0.34; *p* < 0.0001), the HRD cohort (13.6 *vs*. 5.4 months; HR = 0.32; 95% CI, 0.24–0.42; *p* < 0.0001), and the intention-to-treat population (10.8 *vs*. 5.4 months; HR = 0.36; 95% CI, 0.30–0.45; *p* < 0.0001). The benefit was greatest in *BRCA*-mutated patients, followed by the HRD-positive (defined as *BRCA* mutated or LOH-high), HRD-positive (defined as *BRCA* wild-type and LOH-high), and HRD-negative (defined as *BRCA* wild-type and LOH-low) cohorts. ARIEL3 concluded that maintenance rucaparib significantly improves PFS in platinum-sensitive relapsed EOC patients following a complete or partial response to second-line or later platinum-based chemotherapy.

### First-Line Maintenance Therapy With PARP Inhibitor in Newly Diagnosed EOC Patients

In the SOLO1 trial (12), newly diagnosed advanced stage EOC patients with germline or somatic *BRCA* mutations receiving olaparib maintenance therapy had a significant improvement in PFS than the placebo group (median not reached *vs*. 13.8 months; HR = 0.30; 95% CI, 0.23–0.41; *p* < 0.001). In a 5-year follow-up, the median PFS was significantly longer in patients who received olaparib than those who received placebo (56.0 *vs*. 13.8 months; HR = 0.33; 95% CI, 0.25–0.43) (46).

In the PRIMA trial (11), the status of homologous recombination was determined by the *BRCA* mutation or myChoice assay with GIS ≥42. The median PFS was significantly longer in the niraparib group than in the placebo group among the HRD cohort (21.9 *vs*. 10.4 months; HR = 0.43; 95% CI, 0.31–0.59; *p* < 0.001) and the intention-to-treat population (13.8 *vs*. 8.2 months; HR = 0.62; 95% CI, 0.50–0.76; *p* < 0.001). Analyses of GIS in the *BRCA* wild-type cohort was a preplanned exploratory analysis. The results showed a benefit in all *BRCA* wild-type patients regardless of the GIS, though it was higher in the GIS-high subgroup compared with the GIS-low subgroup (HR = 0.5; 95% CI, 0.31–0.83 *vs*. HR = 0.68; 95% CI, 0.49–0.94). PRIMA concluded that niraparib maintenance therapy significantly prolonged the PFS in newly diagnosed advanced stage EOC patients who responded to platinum after primary treatment regardless of homologous recombination status (11).

In the VELIA trial (10), the status of homologous recombination was determined by the *BRCA* mutation or myChoice assay with GIS ≥33. The median PFS was significantly longer in the veliparib-throughout group than in the control group among the *BRCA*-mutated cohort (34.7 *vs*. 22.0 months; HR = 0.44; 95% CI, 0.28–0.68; *p* < 0.001), the HRD-positive cohort (31.9 *vs*. 20.5 months; HR = 0.57; 95% CI, 0.43–0.76; *p* < 0.001), and the overall population (23.5 *vs*. 17.3 months; HR = 0.68; 95% CI, 0.56–0.83; *p* < 0.001). VELIA concluded that a regimen of carboplatin, paclitaxel, and veliparib induction therapy followed by veliparib maintenance therapy leads to significantly longer PFS than carboplatin plus paclitaxel induction therapy alone. The PFS benefit was less pronounced in VELIA than in the other frontline studies, possibly because the HRD cohort contained more false positives, with a higher percentage of patients classified as having HRD (55%) despite a lower percentage of *BRCA-*mutated patients (26%).

In the PAOLA-1 trial (47), the status of homologous recombination was determined by tumor *BRCA* mutations or myChoice assay with GIS ≥42. The median PFS was significantly longer in the olaparib plus bevacizumab group than in the placebo plus bevacizumab group among the overall population (22.1 *vs*. 16.6 months; HR = 0.59; 95% CI, 0.49–0.72; *p* < 0.001), the *BRCA-*mutated cohort (37.2 *vs*. 21.7 months; HR = 0.31; 95% CI, 0.20–0.47), the *BRCA* wild-type cohort (18.9 *vs*. 16.0 months; HR = 0.71; 95% CI, 0.58–0.88), the *BRCA*-mutated HRD-positive cohort (37.2 *vs*. 17.7 months; HR = 0.33; 95% CI, 0.25–0.45), and the *BRCA* wild-type HRD-positive cohort (28.1 *vs*. 16.6 months; HR = 0.43; 95% CI, 0.28–0.66). PAOLA-1 concluded that maintenance olaparib provides a significant PFS benefit in advanced EOC patients receiving first-line standard chemotherapy with bevacizumab, especially in patients with HRD-positive tumors, including those without a *BRCA* mutation. In the HRP cohort, the addition of olaparib to bevacizumab did not improve the PFS (16.6 *vs*. 16.2 months; HR = 0.43; 95% CI, 0.75–1.35). The findings indicate that the GIS has the potential to identify HRD-negative EOC patients who do not derive a benefit from olaparib in combination with bevacizumab (15).

Based on these trials, PARPis were approved by the US FDA and European Medicines Agency (EMA) in the management of EOC patients: (1) salvage monotherapy in *BRCA* mutant or HRD-positive disease after multiple prior lines of therapy, (2) second-line maintenance therapy in recurrent platinum-sensitive disease regardless of *BRCA* mutation or HRD status, and (3) first-line maintenance therapy for newly diagnosed advanced stage platinum-sensitive disease with/without *BRCA* mutation or HRD-positive status (**Table 3**). The homologous recombination status of EOC patients is critical to achieving survival benefits from PARPi treatment. Therefore, how to define the homologous recombination status of EOC patients using HRD assays is an important issue for clinicians.

**Table 3:** FDA and EMA approval of PARP inhibitors in epithelial ovarian cancer.

Approval	PARPi	Indication	Category of therapy	Patient restrictions (*FDA-approved companion diagnostic*)	Evidence
2014 FDA	Olaparib	Recurrent patients after 3 or more lines of chemotherapy	Salvage monotherapy after multiple prior lines of therapy	Germline BRCA mutation (*Myriad BRACAnalysis CDx*)	Study 42
2014 EMA	Olaparib	Maintenance in recurrent patients in complete or partial response to platinum-based chemotherapy	Second-line maintenance monotherapy	High-grade cancers	Study 19
2017 FDA	Olaparib	Maintenance in recurrent patients in complete or partial response to platinum-based chemotherapy	Second-line maintenance monotherapy	No restriction	SOLO-2 Study 19
2017 FDA	Niraparib	Maintenance in recurrent patients in complete or partial response to platinum-based chemotherapy	Second-line maintenance monotherapy	No restriction	NOVA
2017 FDA	Rucaparib	Recurrent patients after 2 or more lines of chemotherapy	Salvage monotherapy after multiple prior lines of therapy	Germline or somatic BRCA mutation (*FoundationFocus™ CDx BRCA*)	ARIEL2
2018 FDA	Olaparib	Maintenance in newly diagnosed advanced stage patients after complete or partial response to first-line platinum-based chemotherapy	First-line maintenance monotherapy	Germline or somatic BRCA mutation (*FoundationOne CDx; Myriad BRACAnalysis CDx*)	SOLO-1
2018 EMA	Rucaparib	Recurrent patients after 2 or more lines of chemotherapy	Salvage monotherapy after multiple prior lines of therapy	Platinum-sensitive relapsed cancers; Unable to tolerate further platinum therapy; Germline or somatic BRCA mutation	ARIEL2
2018 FDA	Rucaparib	Maintenance in recurrent patients in complete or partial response to platinum-based chemotherapy	Second-line maintenance monotherapy	No restriction	ARIEL3
2019 EMA	Olaparib	Maintenance in newly diagnosed advanced stage patients after complete or partial response to first-line platinum-based chemotherapy	First-line maintenance monotherapy	High grade cancers; Germline or somatic BRCA mutation	SOLO-1
2019 FDA	Niraparib	Recurrent patients after 3 or more lines of chemotherapy	Salvage monotherapy after multiple prior lines of therapy	Germline or somatic BRCA mutation; HRD-positive (*Myriad myChoice CDx*)	QUADRA
2020 FDA	Olaparib	Maintenance combined with bevacizumab in newly diagnosed advanced stage patients after complete or partial response to first-line platinum-based chemotherapy	First-line maintenance combined therapy	Germline or somatic BRCA mutation; HRD-positive (*Myriad myChoice CDx*)	PAOLA-1
2020 FDA	Niraparib	Maintenance in newly diagnosed advanced stage patients after complete or partial response to first-line platinum-based chemotherapy	First-line maintenance monotherapy	No restriction	PRIMA

EMA, European Medicines Agency; FDA, Food and Drug Administration; PARPi, PARP inhibitor.

## Homologous Recombination Deficiency Assays

In general, HRD assays are of three main categories: germline or somatic mutations in genes in the HRR pathway, genomic scars or mutational signatures representing patterns of genomic instability, and checking the function of RAD51 localization to sites of DNA damage (18, 21, 34, 48–50).

### Germline or Somatic Mutations in Genes in the HRR Pathway

#### *BRCA1/2* Gene Mutation Tests

*BRCA1/2* mutation tests are well known in the clinical management of EOC patients. The g*BRCA* tests with genetic counseling should be performed near the time of diagnosis for all patients with newly diagnosed EOC (51–53). Both germline and tumor *BRCA* tests can identify EOC patients who would benefit from PARPi maintenance therapy. Notably, a somatic test cannot substitute for a germline test because 5% of g*BRCA*-mutated patients test negative for tumor BRCA. Approximately 11%–18% of patients have a g*BRCA* mutation. The g*BRCA* test not only informs the patient but can also identify family members at risk of possible associated malignancies, which will be helpful for early detection or prevention (51, 52, 54). In patients with negative g*BRCA* tests, somatic *BRCA* tests can identify another 6%–7% of patients with somatic *BRCA* mutations (34, 48, 55–57).

The majority of clinical trials have demonstrated that advanced stage, mainly high-grade serous type, EOC patients with g*BRCA* or somatic *BRCA* mutations derive the greatest benefit from PARPi maintenance therapy in primary first-line management or in platinum-sensitive recurrent disease (10–15, 44, 47). The tumor BRCA status, including germline and somatic *BRCA* mutations, was generally used as a response predictor in these trials (14, 44, 47). A few studies presented data on PARPi treatments only for EOC patients with somatic *BRCA* mutations. The PFS benefit of PARPi compared with placebo in EOC patients with somatic *BRCA* mutations and platinum-sensitive recurrent disease was similar to the benefit in those with g*BRCA* mutations in study 19 (olaparib, HR = 0.23 *vs.* 0.17, respectively) (58), NOVA (niraparib, HR = 0.27 *vs.* 0.27, respectively) (13), and ARIEL2 Part 1 (rucaparib, response rate 74% *vs.* 85%, respectively) (38). For newly diagnosed advanced disease, a similar trend was noted for patients with somatic and g*BRCA* mutations (veliparib, HR = 0.35 *vs.* 0.5, respectively) in the VELIA trial (10). However, patients with *BRCA* wild-type had a smaller, but still significant, benefit from PARPi use in primary first-line management or in platinum-sensitive recurrent disease (13–15). This indicated a poor negative predictive value of the *BRCA* mutation status as a predictor of the PARPi response.

#### Non-*BRCA* Gene Mutation Tests in the HRR Pathway

In addition to *BRCA1/2* genes, non-*BRCA* genes involved in the HRR pathway include *ATM*, *BRIP1*, *NBN*, *PALB2*, *RAD51B*, *RAD51C*, and *RAD51D* (34, 56, 59–62). Patients with germline or somatic mutations in non-*BRCA* HRR genes also derive a survival benefit from DNA repair inhibitors (34, 63–66). However, the prevalence of non-*BRCA* HRR gene mutations is quite low, making it difficult to determine the association of individual genes with clinical outcomes, and all non-*BRCA* HRR gene mutations are usually pooled together for interpretation in these studies. For example, in study 19, 21 platinum-sensitive recurrent patients with non-*BRCA* somatic mutations, including *BRIP1*, *CDK12*, *RAD54L*, and *RAD51B*, derived a similar PFS benefit as those with *BRCA* mutations (HR = 0.21 and 0.18, respectively) (67). In ARIEL2, non-*BRCA* HRR gene mutations were noted in 20 patients (10%), including *ATM*, *BRIP1*, *CHEK2*, *FANCA*, *FANCI*, *FANCM*, *NBN*, *RAD51B*, *RAD51C*, and *RAD54L*. The sensitivity of non-*BRCA* HR gene mutations in discriminating the rucaparib response was only 11%. Furthermore, how many non-*BRCA* HRR genes should be included in the testing list is still unclear. A major challenge for HRR gene testing is the annotation of variants of uncertain significance (VUS) (68–70). In the broader gene panel tests, the functional and clinical impacts of most individual mutations in the genomic loci have not been well characterized. In many cancers, the somatic VUS are more numerous and diverse than germline VUS (71). There is a difference in the reporting rate of *BRCA* VUS (3%–50%), the protocols for detection, and the strategies for management between individual laboratories (72). Currently, the evidence is not sufficient to determine which individual or panel of non-*BRCA* HRR genes could be used to predict the PARPi response.

### Genomic HRD Assays

#### Current HRD Assays: Genomic Instability Score or Percent Genomic LOH

Defects in the HRR pathway generate genetic variations, chromosome structural abnormalities, and genomic instability. These so-called genomic scars or mutational signatures are permanent even if the function of the HRR pathway is restored, which represents a record of DNA damage repair through different pathways in response to harmful exposure in cells (26). Genetic variations generally consist of single nucleotide variants, small insertions and deletions (indels), copy number variations (CNVs), and large chromosomal rearrangements. Single nucleotide variants (SNVs) are base substitutions that may lead to deleterious missense or nonsense mutations. Indels less than 1,000 kbp in length may cause frameshift mutations. CNVs with DNA insertions or deletions more than 1 kb in size may lead to increased expression of oncogenes or decreased expression of tumor suppressor genes. Large chromosomal rearrangements, such as translocations, are a common cause of LOH. The current methods of detecting “genomic scars” use SNP-based microarray technologies to measure somatic CNV, including LOH, telomeric allelic imbalance (TAI), and large-scale state transitions (LSTs) (73). LOH indicates permanent loss of an allele copy in DNA of more than 15 Mb, which renders the tumor cell homozygous at that locus (74). TAI refers to different allele copy numbers of more than 11 Mb in the subtelomere but not crossing the centromere (75, 76). An LST indicates chromosomal breaks between adjacent genomic regions of more than 10 Mb as a result of translocations, copy gains, and copy losses (77). The degree of HRD highly correlates with the LOH, TAI, or number of LSTs in the chromosomes. The GIS combines the information derived from LOH, TAI, and LSTs to represent the degree of genomic instability (76).

The myChoice CDx (Myriad Genetics) and Foundation Focus CDx *BRCA* LOH (Foundation Medicine) are currently the most common commercial assays (78, 79). The myChoice CDx (Myriad Genetics) includes a tumor *BRCA* mutation test and GIS. The Foundation Focus CDx *BRCA* LOH (Foundation Medicine) includes a tumor *BRCA* mutation test and genomic LOH. Both assays use next-generation sequencing (NGS) platforms to identify SNVs, indels, and large rearrangements in genes of tumor tissue (42, 78), and the *BRCA* gene mutation results are concordant in these two assays (67). In the myChoice CDx assay, GIS ≥42 is regarded as “HRD positive” (78). Briefly, tumor tissue is sequenced with a panel of single-nucleotide polymorphisms (SNPs) to generate allele-specific copy number profiles to calculate the sum of LOH, TAI, and LST, resulting in a continuous score from 0 to 100, the GIS. A score of 42 is the 5th percentile in a cohort of ovarian and breast cancers in which HRD was defined as biallelic loss of *BRCA1/2* (78). In the Foundation Focus CDx assay, the percent genomic LOH, calculated as the fraction of genome regions with LOH determined by sequencing SNPs in tumor DNA, is used to measure HRD status. The LOH cutoff value is determined independent of tumor *BRCA* status, and 14% genomic LOH was considered to indicate HRD-positive cells in the ARIEL2 trial (38). In the subsequent ARIEL3, this was adjusted to 16% genomic LOH as the threshold (14). However, the compatibility between HRD defined by the GIS and percent genomic LOH needs to be determined.

FDA has approved companion diagnostic HRD assays for salvage monotherapy in recurrent disease after multiple prior lines of therapy and in first-line maintenance therapy. In the first-line maintenance setting, ESMO suggests that germline and somatic *BRCA* mutation tests are routinely recommended to identify EOC patients who should receive PARPi therapy. A validated HRD assay is reasonable to stratify *BRCA* wild-type EOC patients who would benefit from PARPi therapy (21) although PARPis were approved as maintenance therapy for all platinum-sensitive recurrent EOC patients regardless of *BRCA* mutation or HRD status. The PFS benefits of PARPi maintenance therapy decrease incrementally from *BRCA*-mutated to HRD-positive to HRD-negative cohorts according to the current HRD assays. It may be helpful for the platinum-sensitive relapsed EOC patients to choose bevacizumab or PARPi as maintenance therapy. ESMO suggests that *BRCA* mutation tests and validated genomic scar-based HRD assays are reasonable for predicting the benefits of PARPi use in platinum-sensitive recurrent disease (21). An unmet need is determining whether the HRD assays can consistently identify a subgroup of patients who do not benefit from PARPi therapy. The HRD-negative (*BRCA* wild-type and GIS <42) subgroup constituted approximately 50% of all participants in these trials (10, 11, 47). All of these trials showed a benefit in the intention-to-treat population, which is not sufficient to determine whether the HRD-negative EOC patients benefitted from maintenance PARPi therapy. None of these trials were prospectively designed to stratify the patients into subgroups by HRD assay, and the clinical benefit of PARPi maintenance therapy in HRD-related cohorts was obtained from exploratory analyses.

The cost-effective analysis in platinum-sensitive recurrent EOC showed that maintenance PARPi therapy is the preferred strategy over a treat-all strategy for patients with *BRCA* mutations or in HRD-positive patients (16). In the model, mean costs and progression-free quality-adjusted life years were $827 and 3.4 months for observation, $46,157 and 5.7 months for a *BRCA*-only strategy, $109,368 and 8.5 months for a g*BRCA* and HRD-positive strategy, and $169,127 and 8.8 months for a treat-all strategy. Another cost-effective analysis of “PARPi-for-all” compared with an HRD assay-directed strategy using the models established from the PRIMA, VELIA, and PAOLA-1 trials showed that front-line PARPi maintenance therapy should be reserved for HRD-positive EOC patients until the cost of PARPi is significantly reduced (17). In the analysis, the mean cost per patient for the “PARPi-for-all” strategy was $166,269, $286,715, and $366,506 for the PRIMA, VELIA, and PAOLA-1 trials, respectively. For the HRD assay-directed strategy, the mean cost per patient was $98,188, $167,334, and $260,671 for the PRIMA, VELIA, and PAOLA-1 trials, respectively. However, to the best of our knowledge, a cost-effective analysis between HRD assays is currently lacking for EOC.

The current HRD assays have some limitations (**Table 4**). Multiple causes of discordance between clinical PARPi responses and HRD assay results are possible, including timing, quality and quantity of samples, tumor heterogeneity, reversion mutations, and non-HRD-related PARPi resistance mechanisms. The assays define HRD based on genomic scars, but these assays are unable to represent dynamic changes in the homologous recombination function of the cells. For example, in ARIEL2 (38), pretreatment biopsies and archival tumor biopsies (median time lapse: 2.7 years) were both used for genomic LOH assays in 117 patients. Approximately 34% of patients with LOH-low in the initial archival tissue biopsy turned out to be LOH-high in the pretreatment biopsy, and 30% of these patients had partial responses to rucaparib, which is similar to the LOH-high cohort. Fresh tumor samples may differ from archival tissue samples due to tumor heterogeneity and clonal evolution, and assays using fresh samples more accurately reflect the current status of the tumor (80, 81).

**Table 4:** Limitations of the current HRD assays.

**Technique aspects**
Adequacy and quality of tumor portion of tissue samples Intratumor heterogeneity between specimen biopsy site and other tumor-invasive or metastatic sites Unable to report specific forms of DNA abnormalities or underlying mechanism Define HRD status based on the “genomic scars” but not reflect the current HR functions Indeterminate cutoff threshold to define HRD status
**Tumor aspects**
Intratumor heterogeneity between specimen biopsy site and other tumor-invasive or metastatic sites Unable to report secondary variations in HR genes, especially in archival tumor samples:
- Reversion mutations of *BRCA1/2*, *RAD51C*, *RAD51D*, or *PALB2* - Reverse promotor hypermethylation of *BRCA1* or *RAD51C* - Alternative translation initiation or splicing of *BRCA1*
Unable to detect non-HRR gene-related PARPi resistance:
- Upregulation of membrane drug transporters (e.g., *ABCG*, *MDR1*) - Modulation of DSB end resection - Stabilization of stalled replication forks

A *BRCA* or HRR mutation may form a genomic scar detected by HRD assays, but the tumor could restore the HRR function through reversion or secondary mutations, causing resistance to chemotherapy or PARPis over time (82–84). Reversion or secondary somatic mutations have been identified in *BRCA1/2*, *RAD51C*, *RAD51D*, and *PALB2* in platinum-resistant EOC tumors (85–87). *BRCA* reversion mutations have been identified in 10%–30% of tumors after platinum exposure, which could lead to platinum or PARP resistance (85, 88). The HRD assays are unable to account for reversion mutations because the original gene mutations have resulted in a permanent, irreversible genomic scar. In platinum-resistant EOC patients, the current HRD assays may be inadequate for predicting PARPi responses, as in the QUADRA trial. However, the reversion mutations may be found only in fresh tissue samples and are difficult to detected in germline or archival samples. For tumor suppressor genes, retention of one wild-type allele reduces the effect of a somatic or g*BRCA1/2* mutation (89); however, current HRD assays are unable to report the status of both alleles. In addition, there are other mechanisms of treatment resistance that cannot be detected by HRD assays, including upregulation of membrane drug transporters, modulation of DSB end resection, protected stalled replication forks, and stabilization of the *BRCA1* C-terminal domains (90–94). Intratumor heterogeneity, clonal adaptation, and cancer evolution are challenges for precision medicine (95). The current HRD assays were developed to stratify EOC patients by PARPi benefit and it is uncertain that these assays could be utilized for other inhibitors targeting other genes or molecules involved in the DNA repair pathway. The genomic scar-based HRD assays cannot provide a real-time dynamic evaluation of the current status of the tumor, which may limit the predictive ability, especially when the testing samples are obtained from archival tissue.

#### HRD Assays in Development: Targeted Gene Panel, Whole Exome Sequencing, and Whole Genome Sequencing

Several HRD assays are in development and are summarized in **Table 5**. Tumor NGS assays by targeted gene panel are clinically available mutation tests with panels consisting of variable sizes of select cancer-susceptible genes to identify SNVs, small indels, CNVs, and large chromosomal rearrangements by NGS techniques (96). Larger panels provide more clinically useful information but increase the VUS rates (97). However, mutations or variants identified on tumor NGS assays may not correlate with the clinical response (98). Tumor NGS assays using whole genome sequencing (WGS) or whole exome sequencing (WES) are also in development to assess the tumor HRD status. WGS identifies the whole genome, whereas WES identifies all protein coding regions of tumor DNA, which comprise 1%–2% of the genome but approximately 85% of all disease-causing mutations (99, 100). The raw data generated by WGS is, on average, 30 times larger than the raw data generated by WES; thus, WGS is more time consuming and expensive (101, 102).

**Table 5:** Main strengths and weaknesses of the HRD assays.

Platform	Strengths	Weaknesses
Genomic HRD assay		
*BRCA* mutation + GIS score (or LOH score)	FDA-approved companion test Rapid analysis	Unable to detect non-*BRCA* HRR gene mutations Indeterminate cutoff threshold defining HRD status Intratumor heterogeneity between specimen biopsy site and other tumor-invasive or metastatic sites Unable to represent the functional HR
Targeted gene panel	Customized gene panel More rapid analysis Fewer variant of uncertain significance (VUS) results	Report limited to the customized genes of the panel Unable to detect noncoding and structural variants Heterogeneous coverage based on library preparation and enrichment method Unable to represent the functional HR
Whole exome sequencing	Analysis of all coding exons (2% of genome) Detection of novel somatic mutations	Unable to detect noncoding and structural variants Heterogeneous coverage based on library preparation and enrichment method Unable to represent the functional HR
Whole genome sequencing	Analysis of all coding and noncoding regions in the genome Detect CNV, variants, and structural rearrangements with high sensitivity Mutational signatures: “Signature 3” is associated with BRCA mutations in ovarian cancer	Expensive and time consuming Difficult interpretation of results (much VUS) Unable to represent the functional HR
Promoter methylation	Hypermethylation of *BRCA1* and *RAD51C* in association with HRD has been reported	Unable to detect HRR gene mutations Conflicting results for HRR gene methylation in predicting PARPi response Unresolved technical problems, including sample purity, quantitative protocols, and definition of gene copy number changes Unable to represent the functional HR
Functional HRD assay		
RAD51 foci formation assay	Represents the functional HR	Unable to detect HRR gene mutations Unresolved technical problems, including timing, tissue sampling, DNA damage induction, methods of measurement, and definition of HRD Unsuitable for slowly proliferating tumors Unable to identify defects in *RAD51* downstream or *RAD51*-independent mechanisms
DNA fiber assay	Represents the functional HR	Unable to detect HRR gene mutations Requires fresh, viable tissues Indeterminate definition of replication fork degradation Indeterminate correlation with clinical response

Mutational signatures describe distinct patterns of nucleotide transitions with the context of the surrounding nucleotides from the WGS data of human tumors (71). Different DNA damage processes, such as aging, UV light, radiation, cytotoxic drugs, and DNA repair pathway defects, have been correlated with different patterns of mutations, which can generate characteristic mutational signatures using computational methodologies (103, 104). “Signature 3” is associated with *BRCA1/2* mutations and *BRCA1* promoter methylation in breast, ovarian, pancreatic, and stomach cancers (71, 105, 106). This is a pattern of single-base substitutions in which mutations are distributed among the six possible types (e.g., C > A, C > G, C > T, T > A, T > C, T > G) and the surrounding sequence contexts (bases on either side of the mutated base). Signature 3 has been proposed as a biomarker of HRD, but some challenges make it unsuitable for guiding PARPi therapy in EOC (50, 107). The majority of high-grade serous EOCs contain some contribution from signature 3, and it probably lacks specificity as a HRD biomarker. In addition, signature 3 remains a static representation of HRD on the genome rather than the functional state. HRDetect is a WGS-based assay based on aggregating six HRD-associated signatures into a single score, including microhomology-mediated deletions, base substitution signature 3, rearrangement signature 3, rearrangement signature 5, HRD index, and base substitution signature 8 (105). HRDetect has been shown to predict *BRCA* deficiency with a sensitivity of 98.7% in 560 breast cancers (including the training cohort), 86% in a validation breast cancer cohort (*n* = 80), and nearly 100% in validation ovarian cancer (*n* = 73) and pancreatic cancer (*n* = 96) cohorts. However, the ability of HRDetect to predict the PARPi response in EOC has not been confirmed (108–110). The major limitations of HRDetect include requiring fresh frozen tissue with >50% tumor cells, higher expense, and longer time to carry out the assay (111). Another limitation is that it represents the historical existence of a genomic scar but not the current HRR status.

#### HRD Assays in Development: Gene Promoter Methylation

Promoter methylation results in decreased gene transcription, and an association of gene promoter methylation in *BRCA1* and *RAD51C* with HRD had been reported. However, clinical studies of HRR gene methylation have reported conflicting results, and its role in predicting PARPi responses in EOC patients is controversial (38, 56, 112–115). For example, *BRCA1* promoter hypermethylation was detected in 13% of tumors and *RAD51C* promoter hypermethylation in 2% of tumors in ARIEL2. Genomic LOH (test sensitivity 78%) performed better than non*-BRCA* HRR gene mutations (sensitivity 11%, *p* < 0.001) or promotor hypermethylation (sensitivity 48%, *p* < 0.02) in predicting a response to rucaparib. Some technical problems still need to be resolved, including sample purity, quantitative methylation assays, and gene copy number changes (112, 113, 116–118)

### Functional HRD Assays

#### *RAD51* Foci Formation Assay

In order to define HRD in real time, the tests should theoretically check an important downstream factor integrating multiple upstream HRR pathways to provide a dynamic evaluation of the actual HRD status (119). *RAD51* encodes a recombinase that plays an important role in HRR and replication fork processing (119, 120). In HRR of DSBs, *RAD51* units are localized to the site of the DNA break with the help of numerous mediator protein complexes, including *BRCA1* and *BRCA2*, to facilitate DNA strand invasion into the sister chromatid. The *RAD51* subnuclear foci is generally present after DNA damage in normal cells, but cells with HRD cannot form *RAD51* foci. Therefore, measurement of *RAD51* subnuclear foci is a functional assay for detecting HRD in tumor samples (121, 122).

Studies have demonstrated that the absence or decrease of subnuclear *RAD51* foci correlates with *BRCA* mutations, the response to chemotherapy or PARPi treatment, and clinical prognosis (123–129). Immunofluorescence has been used for evaluation of *RAD51* foci formation in live cells derived from tumors, ascites, or patient-derived xenograft models in which DNA damage was induced by irradiation, platinum, or PARPis (126, 128, 130, 131). Mukhopadhyay et al. reported the first functional assay from live cells/tumor tissue from a prospective series of consecutive EOC patients. The functional assay for HRD using *RAD51* foci formation correctly predicted that approximately 50% of EOC are HRD ahead of the TCGA publication in 2011 (130); the series was subsequently expanded by the same group to show its validity in a various live cell storage and sample transport conditions (132). The homologous recombination Repair CAPacity (RECAP) assay developed by Meijer et al. measures *RAD51* foci in proliferating cells from fresh breast cancer tissue after *ex vivo* radiation. They found a strong correlation between RECAP-defined HRD and *BRCA* mutations. Thus, the RECAP assay could identify HRP tumors that developed *BRCA* reversion mutations in patients with g*BRCA* mutations (133). Immunohistochemistry of *RAD51* foci was demonstrated in formalin-fixed paraffin-embedded (FFPE) samples of breast cancer, and positive *RAD51* foci correlated with resistance to platinum or PARPi (110, 124, 134). Waks et al. found that the positive *RAD51* foci were also associated with *BRCA* reversion mutations, through which the HRR pathway was restored and correlated with platinum resistance (134).

Hill et al. developed another method of checking *RAD51* expression and replication fork stability in organoid cultures (124). The organoids derived from ascites or EOC tumors were checked for the numbers of cells with *RAD51* foci after irradiation. They also stained γH2AX and geminin to mark DNA damage and the S/G2 phase of the cell cycle. The HRD score was generally calculated as the number of *RAD51*-positive cells over the number of cells in S/G2 (marked by geminin or cyclin A2). Samples were defined as HR-low (<10%–20% *RAD51*-positive cells), HR-intermediate (10%–20% to 35%–50% *RAD51*-positive cells), or HR-proficient (>35%–50% *RAD51*-positive cells). However, the correlation of the clinical response with the organoid *RAD51* foci by immunohistochemistry needs to be determined (135).

#### DNA Fiber Assay

The DNA fiber assay was developed to evaluate the dynamics of the replication fork (136–139). The chromosome unzips the intertwined DNA strands to make copies during S phase of the cell cycle, forming the replication fork. The replication fork stops when encountering DNA damage to allow DNA repair before replication continues. Stalled replication forks require numerous proteins, such as BRCA1, BRCA2, and RAD51, to protect from degradation by nucleases. After damage is repaired, the stalled fork resumes its progress. If the damage cannot be repaired to resume DNA synthesis, the stalled replication fork undergoes degradation, with the fork collapse leading to cell apoptosis (140–143). Extensive replication fork degradation is associated with chemosensitivity in *BRCA-*mutated tumors (91, 119). Briefly, the DNA fiber assay evaluates the dynamics of replication forks by incorporating DNA with two labeled thymidine analogs, iododeoxyuridine (IdU) and chlorodeoxyuridine (CldU), which can be visualized by an immunofluorescence-based approach (139, 144, 145). Degradation of the stalled forks leads to shortening of the thymidine-labeled tract (124, 144, 145), and it is commonly detected in the absence of key factors, such as *BRCA1* or *BRCA2* (91, 146–148). A preclinical study showed that DNA fiber assays correlate better with platinum sensitivity than PARPi sensitivity (124). Further studies are needed to verify the feasibility of DNA fiber assays in predicting PARPi sensitivity.

The challenges with the *RAD51* foci assay include exam timing (pre- or posttreatment), tissue resources (primary tumor, ascites, or FFPE), methods of inducing DNA damage (radiation, platinum, or PARPi), methods of detection (immunofluorescence or immunohistochemistry), measurement of *RAD51* foci (numbers or percentages), intratumor or interobserver variability, and the definition of HRD in correlation with the clinical response (125, 126, 149). In addition, *RAD51* foci assays are not suitable for slowly proliferating tumors and are unable to identify defects in the HRR pathway downstream of *RAD51* or *RAD51*-independent mechanisms (150–155). The challenges of DNA fiber assays include requiring fresh viable tissues, the definition of replication fork degradation, and the correlation with clinical responses (135). The *RAD51* foci assay and DNA fiber assay are both potential functional HRD assays, but when the commercial assays will be available is unclear.

## Conclusion

PARPi maintenance therapy has made great progress in the management of EOC, with the successful translation of the concept of “synthetic lethality” into clinical practice. PARPis are expensive, and the cost-effective analysis showed that PARPi therapy for *BRCA-*mutated or HRD-positive EOC patients is a preferable strategy over a treat-all strategy. Although FDA-approved companion HRD assays are available for PARPi use, an important unmet problem is that the current HRD assays are unable to consistently identify a subgroup of patients that does not benefit from PARPis, which will lead to increasing medical expenses and possible resistance to PARPi therapy. There are still some issues that need to be resolved, including the quality of tissue samples, intratumor heterogeneity, the cutoff threshold for the definition of HRD, functional HRD status, reversion or secondary mutations of HRR genes, discordance with the clinical response, and cancer evolution. Ongoing development of new comprehensive HRD assays, such as WES/WGS-based assays or functional *RAD51* foci/DNA fiber assays, will improve our ability to select appropriate EOC patients who benefit from PARPi. The comprehensive genomic scar assays based on WGS or WES could provide the HRR gene mutations, mutational signatures, and reversion mutations simultaneously. For tissue retrieval, multistep sampling to obtain archival and pretreatment tumor tissues has the potential to disclose the cancer evolutionary changes over the clinical course to guide the therapy. For precision medicine, it is necessary to develop a comprehensive model integrating the clinical factors, genomic mutational signatures, and functional tests to provide both past evidence of HRD and the current functional ability of HRR. Prospectively designed head-to-head comparison between the various HRD assays incorporating into clinical trials of PARPi monotherapy or in combination with traditional chemotherapy, antiangiogenetic agents, checkpoint inhibitors, or other DNA repair inhibitors is important to establish the optimal clinical implications of HRD assays.

## Author Contributions

Conception and study design: Y-CC and W-FC. Writing and revision of the manuscript: Y-CC, P-HL, and W-FC. Study supervision: W-FC. All authors contributed to the article and approved the submitted version.

## Funding

This work was supported by research grants from National Taiwan University Hospital (NTUH. 105-N02, NTUH. UN105-059, and NTUH. 108-S4230).

## Conflict of Interest

The authors declare that the research was conducted in the absence of any commercial or financial relationships that could be construed as a potential conflict of interest.

## Publisher’s Note

All claims expressed in this article are solely those of the authors and do not necessarily represent those of their affiliated organizations, or those of the publisher, the editors and the reviewers. Any product that may be evaluated in this article, or claim that may be made by its manufacturer, is not guaranteed or endorsed by the publisher.
